# Assessment of Bioavailability after In Vitro Digestion and First Pass Metabolism of Bioactive Peptides from Collagen Hydrolysates

**DOI:** 10.3390/cimb43030113

**Published:** 2021-10-13

**Authors:** Christina E. Larder, Michèle M. Iskandar, Stan Kubow

**Affiliations:** School of Human Nutrition, McGill University, Ste-Anne-de-Bellevue, QC H9X 3V9, Canada; christina.larder@mail.mcgill.ca (C.E.L.); michele.iskandar@mcgill.ca (M.M.I.)

**Keywords:** bioavailability, digestion, bioactive peptides, first pass metabolism, collagen hydrolysate, cell culture, capillary electrophoresis, human intestinal epithelial cells (HIEC-6), permeability

## Abstract

Collagen hydrolysates (CHs) are composed of bioactive peptides (BAPs), which possess health enhancing properties. There is a knowledge gap regarding the bioavailability of these BAPs that involves intestinal transport and hepatic first pass effects. A simulated gastrointestinal model was used to generate digesta from two CHs (CH-GL and CH-OPT), which were applied to a novel transwell co-culture of human intestinal epithelium cell line-6 (HIEC-6) and hepatic (HepG2) cells to simulate in vivo conditions of absorption and first pass metabolism. Peptide transport, hepatic first pass effects, and bioavailability were determined by measuring BAPs (Gly-Pro, Hyp-Gly, Ala-Hyp, Pro-Hyp, Gly-Pro-Hyp) using an innovative capillary electrophoresis method. All peptides were transported across the intestinal cell layer to varying degrees with both CHs; however, Gly-Pro-Hyp was transported only with CH-GL, but not CH-OPT. Notable hepatic production was observed for Ala-Hyp with both CH treatments, and for Pro-Hyp and Gly-Pro with CH-GL only. All peptides were bioavailable (>10%), except for Gly-Pro-Hyp after CH-OPT. Overall, a high degree of transport and hepatic first pass effects on CH-derived BAPs were observed. Further research is needed to explore the hepatic mechanisms related to the production of BAPs and the bifunctional effects of the bioavailable BAPs noted in this study.

## 1. Introduction

Collagen hydrolysates (CHs) have been shown to provide multiple health benefits, which have been primarily attributed to their bioactive peptide (BAP) content [1,2,3]. These BAPs can be found in the hydrolysate products, although an increase in the diversity and content of peptides can result from gastrointestinal (GI) digestion [4,5]. The BAPs released after the digestion of collagen products, such as Pro-Hyp and Gly-Pro-Hyp, can possess multiple health properties, which include antimicrobial and antihypertensive effects, regulating inflammation, reducing pain associated with osteoarthritis, promoting bone synthesis, stimulating wound healing, as well as antioxidant properties and angiotensin-I-converting enzyme inhibitory effects [3,4,6,7].

After digestion, BAPs undergo first pass metabolism, a process defined by hepatic metabolism of compounds following their absorption at the level of the intestinal epithelium that mediates entry into the systemic circulation [8,9]. The bioactivity of BAPs depends heavily on their ability to reach the general circulation intact after oral ingestion, otherwise called bioavailability [9]. Clinical studies have consistently shown that peptides generated from orally ingested collagen precursors, such as gelatin, or collagen hydrolysates, can reach the systemic circulation and be excreted in the urine [4,6,10,11,12]. Importantly, the clinical efficacy of CHs has been demonstrated in multiple trials showing reduction of joint discomfort in athletes with functional knee problems and decreased joint pain in osteoarthritis patients [1,3,13]. The BAPs in the bloodstream identified after oral ingestion of CHs and CH precursors, include Ala-Hyp, Pro-Hyp and Gly-Pro-Hyp [4,6,10,14].

The assessment of peptide bioavailability using human trials remains costly, lengthy and with limited experimental options for sampling due to ethical restrictions. Instead, animal studies have been used to estimate the bioavailability of BAPs from collagen and collagen precursor products [14,15,16,17]; however, predictions of bio-absorbability do not always align with human clinical data due to species differences in intestinal permeability and metabolic activity [2,18]. Bioavailability studies of food components and pharmaceuticals using animal models have demonstrated poor correlations between rats and humans (r^2^ = 0.18) as well as dogs and humans (r^2^ = 0.19) [18]. Due to such species differences in intestinal permeability and metabolic activity, intestinal cell culture models, rather than animal models, are often used to assess the intestinal transport of food-derived BAPs [2].

Caco-2 cells, a human colon carcinoma cell line, has been used regularly to assess for small intestinal (SI) permeability [2]. Previous work by Feng et al. (2017) [19] used the Caco-2 model to estimate the transepithelial peptide transport efficiency of bovine CHs. The bioavailability of the CHs, as determined by amino acid (AA) transport, ranged between ~15 and 23%, depending on the hydrolysis method used to generate the CH. Recent work by Song et al. (2020) assessed the bioavailability of BAPs from silver carp skin hydrolysate using in vitro digestion and Caco-2 cells [7]. They found that, using high-performance liquid chromatography–electrospray ionization tandem mass spectrometry (HPLC-ESI-MS), the transport (%) of Hyp-Gly, Hyp-Gly-Glu and Pro-Gly-Glu-Hyp-Gly was 22.63 ± 5.19, 11.15 ± 0.52 and 18.35 ± 1.20, respectively.

Although in vitro intestinal permeability measures have typically used Caco-2 cells, peptide bioavailability assessments using this cell culture model are not ideal due to the under-expression of peptide transporters such as peptide transporter 1 (PepT1) in these tumorigenic cells. Hence, depending on the compound being assessed, permeability results using Caco-2 cells do not always correlate with human intestinal permeability [18,20]. PepT1, otherwise known as SLC15A1, is the main transporter for di- and tri-peptides, which are predominant in CHs and have been indicated to be primarily responsible for the CH-mediated bioactivities [7,10,15]. To overcome the limited PepT1 expression in Caco-2 cells, a non-tumorigenic human small intestinal epithelial cell (HIEC) line can be used. HIEC cells have been shown to be a superior alternative to Caco-2 cells for predicting transporter-mediated absorption of compounds in humans when taken orally [21,22]. The HIEC cell model also more accurately represents the physiological in vivo conditions of the SI [22,23,24]. To the best of our knowledge, no study has investigated the transport of CH-derived BAPs using HIEC cells. One study investigating salmon protein hydrolysate peptides and their regulation of oxidative protective genes was investigated using HIEC cells; however, no analysis of peptide bioavailability was completed [25].

Methods to accurately quantify di- and tri-peptides to determine their bioavailability have been lacking. Using plasma samples from clinical studies, quantification methods of BAP bioavailability are often calculated using an indirect calculation of Hyp-containing peptides and/or AAs [4,10,14]. Cell culture models also suffer from such limitations in terms of peptide analysis. Feng et al. (2017) assessed the bioavailability of bovine CHs involving Caco-2 cells using an indirect calculation based on the total AAs transported [19] but peptides were not identified or measured. In the present study, our novel method for targeted BAP quantification using capillary electrophoresis (CE) [26,27] was adapted for cell culture media to determine peptide content.

Another limitation to previous in vitro studies investigating BAP bioavailability has been the sole use of intestinal cell cultures without consideration of the subsequent hepatic first pass effects on the intestinally transported BAPs. Some reports have used liver cell culture models, often using human hepatocellular carcinoma (HepG2) cell line, to assess the hepatic metabolism of xenobiotics and drug transporters [8,28]. Previous work has also shown that Pro-Gly can increase PepT1 expression in HepG2 cells, although no assessment of the hepatic effects on Pro-Gly was investigated [29]. Previous studies from our laboratory have assessed the bioavailability of dietary components using a Caco-2/HepG2 co-culture model of first pass metabolism by applying digests from a human simulated gut digestion model [8]. Similar in vitro models have assessed the oral bioavailability of compounds, such as xenobiotics, and have shown very good correlations with in vivo data from humans and animal models [30,31]. In general, there is a major gap in the literature with respect to the study of the hepatic first pass effects on BAPs following their intestinal cell absorption.

In this study, a combination of in vitro gut digestion together with HIEC-6/HepG2-mediated transport and metabolism was used to investigate the bioavailability of BAPs generated after CH digestion. Direct quantification of BAP bioavailability was performed using CE. The aim of this study was to use this novel combination of techniques and cell lines to improve our understanding of the bioavailability and metabolism of CH-derived BAPs that have postulated health promoting properties.

## 2. Materials and Methods

### 2.1. Peptide Standards

Peptide standards Gly-Pro, Hyp-Gly, and Ala-Hyp were ordered and synthesized by CanPep Inc. (Montreal, QC, Canada). Peptides Gly-Pro-Hyp (4008512) and Pro-Hyp (4001630) were purchased from Bachem (Hauptstrasse, Bubendorf, Switzerland). Peptides were 98% pure with peptide purification validation completed by HPLC and mass spectra analysis, provided by the suppliers.

### 2.2. Cells

HIEC-6 (ATCC^®^ CRL-3266™) and HepG2 (ATCC^®^ HB-8065™) cells were purchased from American Type Culture Collection (ATCC, Manassas, Virginia, USA). HIEC-6 cells were cultured using OptiMEM 1 Reduced Serum Medium (Thermo Fisher Scientific, Gibco No. 31985, Waltham, MA, USA) with 20 mM HEPES, 10 mM GlutaMAX (Thermo Fisher Scientific, Gibco No. 35050, Waltham, MA, USA), 10 ng/mL Epidermal Growth Factor, and 4% fetal bovine serum (FBS). HepG2 cells were grown using ATCC-formulated Eagle’s Minimum Essential Medium (Thermo Fisher Scientific, Gibco No. 30-2003, Waltham, MA, USA), with 10% FBS. Cells were maintained at 37 °C with 90% relative humidity and 5% CO_2_ in culture medium.

### 2.3. Treatments

Two bovine-sourced CH products were used in this study: Genacol Original Formula^®^ (Blainville, QC, Canada) (CH-GL) and Selection (Uniprix, QC, Canada) (CH-OPT).

### 2.4. Simulated Digestion

Simulated human digestion was completed to provide digests for first pass metabolism studies in cell culture (see Section 2.6). Upper intestinal digestion involving the stomach and SI was adapted from Alemán et al. (2013), Miranda et al. (2013) and Larder et al. (2021) [5,32,33]. Based on a previous clinical study using CH-GL [13] and previous in vitro digestion models [5], 1200 mg of CHs were digested in reactor vessels placed in a water bath (Cole-Parmer Advantec, TBS181SA, Montreal, QC, CN) at 37 °C, and mounted on a stir plate (Corning, hot plate laboratory stirrer PC351, Corning, NY, USA), where the pH was monitored and adjusted throughout digestion (Fisher Scientific, S90528, Waltham, MA, USA). A 4% *w*/*w* pepsin solution (Sigma-Aldrich, P7125, St. Louis, MO, USA) prepared in 0.1 M HCl was added, and the pH of the solution adjusted to 2. The solution was incubated for 30 min. Afterwards, a 4% *w*/*w* pancreatin solution (Sigma-Aldrich, P7545, St. Louis, MO, USA) was added. The pH was adjusted to 8 and the solution incubated for 2 h. To stop the enzymatic processes, the resulting digesta were rapidly cooled on ice and the pH increased to 10. Digesta were then frozen at −20 °C for temporary storage, until the digesta were filtered using a membrane filter with a molecular weight cut off (MWCO) of 10 kDa in a stirred Amicon ultrafiltration membrane reactor at 4 °C and under nitrogen gas pressure of 40 psi [34]. The filtrates were freeze-dried at −50–−60 °C and 0.85 mBar (0.64 mm Hg) (Gamma 1–16 LSC, Christ, Osterode am Harz, Germany) and stored at −80 °C until used in cell culture. Three independent digestions were completed for each CH treatment.

### 2.5. 3-(4,5-dimethylthiazol-2-yl)-2,5-diphenyl Tetrazolium Bromide (MTT) Assay

HIEC-6 cells were seeded in a 24-well plate at a density of 1 × 10^5^ cells/well and maintained as described above (Section 2.2). Once confluent, the 3-(4,5-dimethylthiazol-2-yl)-2,5-diphenyl tetrazolium bromide (MTT) assay was performed [35]. Cells were incubated for 3 h with a 0.5 mg/mL thiazolyl blue tetrazolium bromide (Sigma-Aldrich, M5655, St. Louis, MO, USA) solution made in phosphate buffer solution. Afterwards, a lysis solution (0.4 N HCl in 100% isopropanol) was added to dissolve the purple formazan crystals that were produced by viable and metabolically active cells. The absorbance was measured at 570 nm and cell viability expressed as survival (%) of untreated cells.

### 2.6. Co-Culture

**Figure 1:**
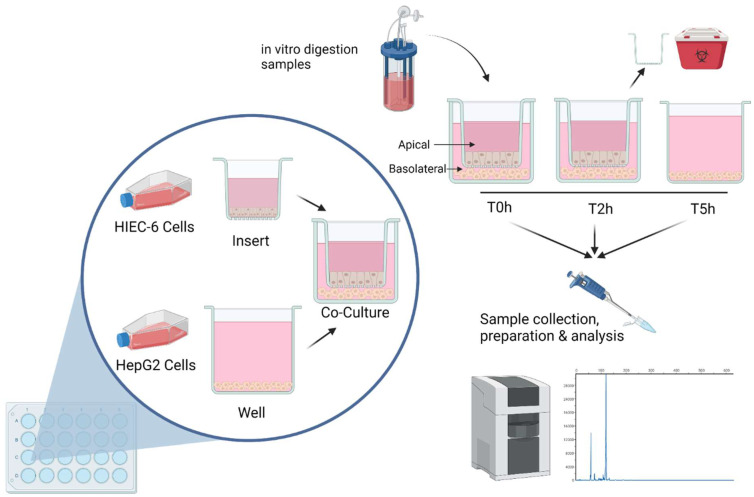
Assessment of first pass metabolism in cell culture. HIEC-6 and HepG2 cells were seeded in a 24-well transwell plate. Freeze-dried gastrointestinal digesta from a simulated digestion model were applied to the apical compartment of the co-culture and incubated for 2 h. The transwell insert was removed and the incubation continued for another 3 h. Subsamples from the apical and basolateral side were taken at times 0, 2 and 5 h, followed by peptide analysis using capillary electrophoresis. Figure created with BioRender.com.

A HIEC-6/HepG2 cell co-culture system was used to determine the bioavailability of targeted BAPs from CHs after digestion (Figure 1). HIEC-6 cells and HepG2 were cultured separately but then later combined in a transwell system using polyester (PET) ThinCerts (Greiner Bio-One, Cat no. 662641, Monroe, NC, USA) and corresponding 24 multiwell cell culture plates (Greiner Bio-One, Cat no. 662160, Monroe, NC, USA). The co-culture methods were adapted from Sadeghi Ekbatan et al. (2018) and Takenaka et al. (2016) [8,22]. HIEC-6 cells were seeded onto ThinCerts at 1 × 10^5^ cells/well. The medium was changed every 2 days and cells were grown for a total of 8–9 days. Transepithelial electrical resistance (TEER) was measured using a volt-ohmmeter to assess the integrity of the monolayer and experiments were conducted when the TEER reached 100 ohm/cm^2^, which has been shown to be appropriate for HIEC-6 cells [22]. HepG2 cells were then added to the basolateral side of the transwell (1 million cells/mL). Preliminary studies in terms of cell viability were completed using MTT to assess for optimal peptide dose range (see Section 2.5). At time 0, the apical medium was replaced with media containing 2 mg/mL reconstituted freeze-dried (FD) CH digesta (either CH-GL and CH-OPT), or only media (blank). The co-culture and treatments were incubated for 2 h at 37 °C, 5% CO_2_. After 2 h, the inserts containing HIEC-6 cells were removed, and the plates containing HepG2 cells were incubated for another 3 h. Samples were taken from the apical and basolateral sides at times 0, 2 and 5 h, and microcentrifuged at 2000 rpm for 15 min. The supernatant was collected and used for subsequent peptide analysis (see Section 2.7). Three independent experiments assessing bioavailability were completed. Controls included inserts without seeded cells (TEER control) and seeded wells with no CHs treatment (only media; negative control).

### 2.7. Targeted Peptide Quantification Using Capillary Electrophoresis (CE)

Peptide analysis was completed using an adapted protocol from Larder et al. (2018) and Larder et al. (2021) (submitted) [26,27]. Samples were purified from cellular and protein debris by adapting the use of Amicon^®^ Ultra-0.5 Centrifugal Filter Devices (Millipore, UFC501096, Burlington, Massachusetts, USA). Samples from cell culture were processed as per the manufacturer’s instructions, however, the filtrate (comprising of peptides) was not discarded and instead used for analysis. A CE system (Capel 205M; Lumex Instruments, Fraserview Place, BC) was used for the targeted quantification of 5 peptides (Gly-Pro, Hyp-Gly, Ala-Hyp, Pro-Hyp, Gly-Pro-Hyp). The instrument was set for 20 °C and the separation capillary (Molex, 2000019, Lisle, Illinois, US) was similar to previous CE methods for collagen analysis [36]; 60 cm in total length, 53 cm effective length, and 75 µm inside diameter. Injections were completed using pressure (30 mbar for 10 s) at 0 kV and analysis was completed at 20 kV using 0 mbar for 1199 s at 205 nm. A 0.1 M phosphate buffer (pH 2.4) was used for rinsing and as running buffer. Filtered samples were diluted with running buffer before injection. Before sample injection, the capillary was rinsed with MilliQ water, 0.5 M NaOH and running buffer, each for 5 min. The electropherograms were processed to determine peak area using the software Elforun (Lumex Instruments Canada, Version 4.2.4, Mission, BC, Canada). Quantification of each peptide, based on peak area, was performed using external standards and corresponding calibration curves, where the linearity was assessed by the coefficients of determination, R^2^. The mean of three measurements for each treatment was taken. Previous CE method papers have also utilized three measurements [37].

The apparent permeability coefficient (P_app_) was calculated similarly to Song et al. (2020) [7], using the standard equation:

P_app_ = ΔQ/(Δt × A × C_0_)

where Δt is the incubation time (s), A is the surface area of the insert filter membrane (cm^2^), C_0_ is the initial concentration of peptides in the apical compartment at time 0 h (μM), and ΔQ is the amount of peptide transported within a given period (μmol/s). The incubation timepoint (Δt) used was representative of the intestinal transport phase (2 h timepoint). Data is reported as mean ± SEM. An assessment of the basolateral compartment at time 0 h showed no peptide presence. Therefore, it was assumed that for each well, treatment and plate, the peptide content off the basolateral compartment at time 0 h was 0.

Transport (%) was assessed using the same equation as Song et al. (2020) [7]. It is a fraction of the amount of transported peptide in the basolateral compartment compared to the initial apical compartment peptide content.

Transport (%) = Transported peptide content (Basolateral 2 h)/Initial peptide content (Apical 0 h) × 100

Hepatic first pass effect (%) was calculated as:

Hepatic effect (%) = Peptide content after incubation with HepG2 (Basolateral 5 h)/Content of peptide available for liver metabolism (Basolateral 2 h) × 100

Bioavailability, after first pass metabolism, was expressed as a percentage of final and initial peptide digesta values, as described in Sadeghi Ekbatan et al. (2018) [8].

Bioavailability (%) = Peptide content after HepG2 incubation (Basolateral 5 h)/Initial amount of peptide (Apical 0 h) × 100

### 2.8. Statistical Analysis

For each peptide, a t-test was completed to assess differences between CH treatments in terms of peptide transport, hepatic effect, and first pass metabolism, where differences were considered significant if *p* < 0.05. MTT was assessed using a two-way ANOVA using dose and treatment as factors, followed by Tukey-HSD. Differences were considered significant if *p* < 0.05. All analyses and figures were completed using GraphPad Prism (Version 9.0.1 for Windows, GraphPad Software, San Diego, CA, USA). Data is reported at mean ± SEM.

## 3. Results

Two bovine-sourced CHs (CH-GL and CH-OPT) underwent simulated human digestion. Filtered digests were applied to a HIEC-6/HepG2 co-culture in a transwell system to determine the transport, hepatic first pass effects and bioavailability of BAPs (Gly-Pro, Hyp-Gly, Ala-Hyp, Pro-Hyp, Gly-Pro-Hyp).

### 3.1. MTT Assay

**Figure 2:**
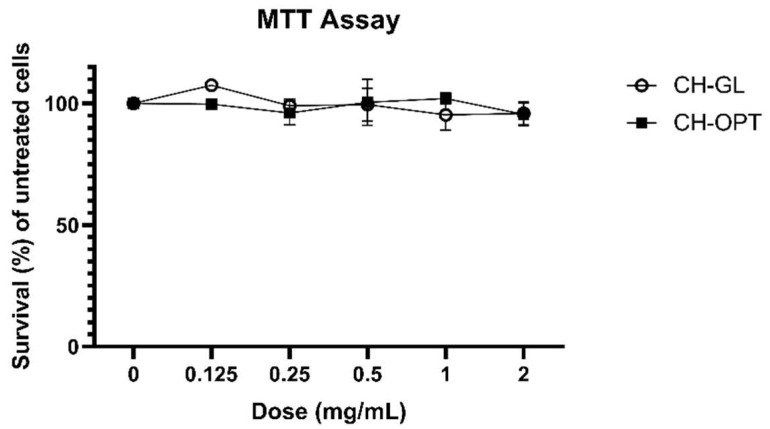
Cell survival (%) using (3-[4,5-dimethylthiazol-2-yl]-2,5-diphenyl tetrazolium bromide) (MTT) method on HIEC-6 cells. A two-way ANOVA, using dose and treatment as factors, followed by Tukey-HSD was completed where differences were considered significant if *p* < 0.05. No significant differences between CH doses or treatments were observed.

Before CH treatments were applied to the HIEC-6/HepG2 co-cultures, a dose response study to assess possible cytotoxicity of the CH treatments was completed (Figure 2). Cell survival was not significantly different between the control (0 mg/mL) and any of the peptide doses (0.125, 0.25, 0.5, 1, 2 mg/mL) for either CH treatment. This work verified that up to 2 mg/mL of reconstituted peptides from simulated CH digestion caused no adverse cytotoxic effects on HIEC-6 cells. The assessment of cytotoxicity helped establish the dose used for subsequent bioavailability studies, as a dose large enough was required to ensure that BAPs would be quantifiable after first pass metabolism.

### 3.2. Peptide Transport

Upper intestinal digests of CHs (CH-GL and CH-OPT) were applied to a HIEC-6/HepG2 transwell co-culture. Samples were collected from the apical and basolateral compartments at time 0 h and after 2 h to determine peptide transport (%) across the intestinal epithelium and apparent permeability (P_app_). After 2 h, sub-samples were again collected, the insert containing HIEC-6 cells was discarded, and the hepatic cells allowed to incubate for another 3 h to determine the hepatic effects on CHs peptides. Samples were taken at the final timepoint (5 h) from the basolateral compartment. No detectable peptide content for either cell culture compartment at any timepoint was observed using the cell culture blank (i.e., no CH added, negative control) (data not shown).

After CH-GL treatment (2 h), 59.44 ± 11.32% of Gly-Pro-Hyp was transported across the intestinal HIEC-6 layer (Table 1). No observable content of Gly-Pro-Hyp was measured in the basolateral compartment of the transwell system after CH-OPT. Transport across the intestinal epithelium was observed for all other peptides (Gly-Pro, Hyp-Gly, Ala-Hyp, and Pro-Hyp) for both CHs. The peptide and treatment with the greatest transport (%) was Hyp-Gly after CH-OPT treatment (82.53 ± 36.53). The greatest transport (%) for CH-GL was also observed with Hyp-Gly (62.41 ± 11.11). The peptides with the least transport (%) were Ala-Hyp after CH-GL (9.27 ± 2.49) and Pro-Hyp after CH-OPT (24.15 ± 1.42).

No differences in peptide transport (%) across the epithelial layer were observed between treatments (CH-GL and CH-OPT) for any of the di-peptides (Gly-Pro, Hyp-Gly, Ala-Hyp, and Pro-Hyp).

The apparent permeability coefficients (P_app_) were also assessed (Figure S1). Similar to the transport (%) results, the peptide Hyp-Gly had the greatest P_app_ compared to all the other di-peptides assessed, for both CH treatments. Specifically, P_app_ (cm/s) for CH-GL was 6.740 ± 1.200 × 10^−6^ and CH-OPT was 5.593 ± 2.476 × 10^−6^. The peptide with the lowest P_app_ was Ala-Hyp, where CH-GL was 0.725 ± 0.195 × 10^−6^ cm/s and CH-OPT was 1.033 ± 0.226 × 10^−6^ cm/s.

No differences in P_app_ were observed between treatments (CH-GL and CH-OPT) for any of the di-peptides. In contrast, P_app_ was measurable for Gly-Pro-Hyp after CH-GL treatment, but no apparent permeability coefficient could be determined for CH-OPT, due to a lack of quantifiable peptide content in the basolateral compartment after 2 h.

### 3.3. Hepatic First Pass Effects

Hepatic first pass effects were observed for the peptide Pro-Hyp (Table 2). An increase in Pro-Hyp following hepatic production by HepG2 cells after CH-GL (151.4 ± 24.3%) compared to CH-OPT (63.63 ± 8.63%) was observed. The peptides Ala-Hyp (304.9 ± 57.2%) and Gly-Pro (109.2 ± 9.6%) increased following hepatic production by HepG2 cells after CH-GL. An increase in Ala-Hyp content was also observed following hepatic production after CH-OPT treatment (198.0 ± 107.6%), although not for Gly-Pro (86.12 ± 14.09%). Hyp-Gly following hepatic action was the least affected (55.16 ± 16.01% after CH-GL and 28.23 ± 6.55% after CH-OPT) compared to the other di-peptides.

There were no differences in hepatic production or metabolism between treatments (CH-GL and CH-OPT) for Gly-Pro, Hyp-Gly, and Ala-Hyp. No hepatic first pass effects for Gly-Pro-Hyp were seen with CH-OPT, as no peptides were transported by the intestinal layer to be available for hepatic action.

### 3.4. Peptide Bioavailability

**Figure 3:**
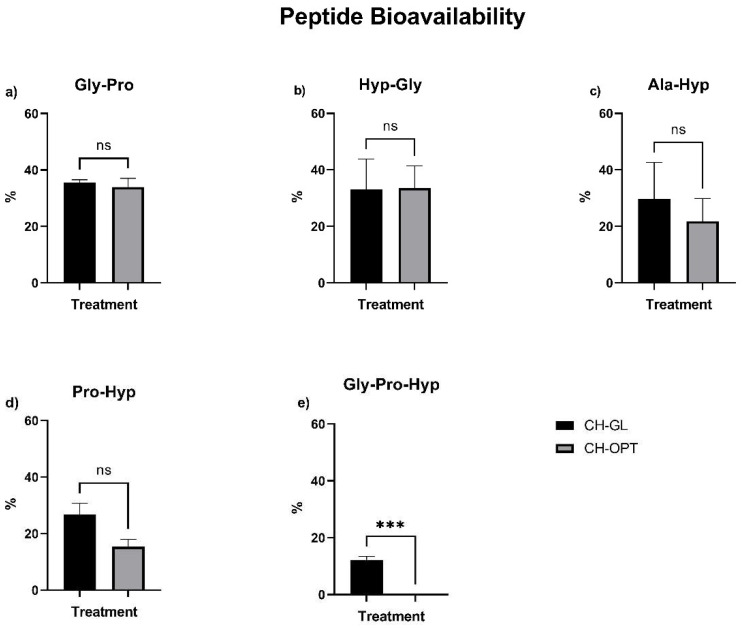
Bioavailability of CH-GL and CH-OPT peptides after first pass metabolism: (**a**) Gly-Pro; (**b**) Hyp-Gly; (**c**) Ala-Hyp; (**d**) Pro-Hyp; and (**e**) Gly-Pro-Hyp. Values are expressed as the final peptide content after hepatic effect as a percentage of initial digesta values. For each peptide, a *t*-test was completed to determine the effect of CH treatment, where differences were considered significant if *p* < 0.05. Columns with asterisks are significantly different (*** *p* < 0.001). Columns with ns are not significantly different.

The bioavailability of the CH-GL and CH-OPT peptides after first pass metabolism was calculated in terms of a percentage of the peptide content observed after hepatic first pass effects when compared to the initial digesta peptide values. Peptide bioavailability was >32% for Gly-Pro and Hyp-Gly after both CH treatments (Figure 3). Ala-Hyp showed an average bioavailability of >20%. Although the bioavailability of Pro-Hyp after CH-GL treatment (26.81 ± 3.97%) appeared to be greater than CH-OPT (15.43 ± 2.60%), this difference did not reach statistical significance (*p* = 0.0745).

The bioavailability of the di-peptides Gly-Pro, Hyp-Gly, and Ala-Hyp after first pass metabolism did not differ between CH treatments. As no tri-peptide content was detected after intestinal transport using CH-OPT treatment, this peptide did not undergo detectable first pass metabolism. After CH-GL treatment, the bioavailability of Gly-Pro-Hyp was 12.24 ± 1.12%.

## 4. Discussion

This work was the first to utilize a HIEC-6/HepG2 co-culture to predict the bioavailability of BAPs after the digestion of two CHs using an optimized CE method. This novel combination of cell lines provided further insight into the high degree of BAP transport by utilizing HIEC-6 cells, which more accurately represents the physiological in vivo conditions than previously utilized Caco-2 cells. In terms of the key observations related to di-peptide transport, the P_app_ for all the di-peptides measured for both CHs were between 1 and 10 × 10^−6^ cm/s. Previous work, establishing the relationship between in vitro (P_app_) and in vivo absorption, have ranked compounds as poorly, moderate, or well absorbed to corresponding P_app_ ranges [7,38]. Poorly absorbed compounds are below 1 × 10^−6^ cm/s, moderately between 1 and 10 × 10^−6^ cm/s, and well absorbed compound are above 10 × 10^−6^ cm/s. Thus, the di-peptides measured in the present study can be considered moderately bioavailable, except for Ala-Hyp after CH-GL treatment, which was 0.7254 ± 0.1947 × 10^−6^ cm/s. It is possible that the moderate and high degree of bioavailability of collagen-derived BAPs are related to the clinically significant health benefits associated with CH intake.

A relatively high (59%) monolayer transport of Gly-Pro-Hyp with a P_app_ value of approximately 9 × 10^−6^ cm/s was noted after CH-GL treatment. The P_app_ of Gly-Pro-Hyp observed with the CH-GL treatment could thus be in the range of a moderately to well absorbed compound. The above P_app_ value was much greater than previously reported for Gly-Pro-Hyp by Sontakke et al. (2016), who using Caco-2 cells followed by LC-MS/MS analysis, showed relatively low cumulative amounts of the tri-peptide transported across the monolayer with a P_app_ value of 1.09 ± 0.03 × 10^−6^ cm/s [15]. The Gly-Pro-Hyp peptide exhibits multiple health promoting properties, most notably inhibition of dipeptidylpeptidase-IV (DPP-IV) [39]. In patients with type 2 diabetes, DPP-IV inhibitors are used to control postprandial glycemia [39]. Future work is needed assessing the in vivo bioavailability and health modulating properties of this peptide in association with the CH-GL treatment.

In the present work, a markedly lower degree of transport for Pro-Hyp (P_app_ = 1.912 ± 0.4794 × 10^−6^) as compared to Gly-Pro-Hyp was observed with the CH-GL treatment. Similarly, the apparent permeability reported by Sontakke et al. (2016) for Pro-Hyp (0.13 ± 0.03 × 10^−6^ cm/s) was significantly lower than their value for Gly-Pro-Hyp [15]. The P_app_ of Pro-Hyp observed in the present study, however, was greater than the values reported by Sontakke et al. (2016) [15] and Feng et al. (2017) (1.45 ± 0.17 × 10^−6^ cm/s) [40]. As noted by the above, the permeation of Gly-Pro-Hyp was greater than Pro-Hyp, even though Gly-Pro-Hyp is a larger molecular weight peptide. Peptide transport across the intestinal layer via paracellular pathways is primarily dependent on the charge and molecular size of the compound. Since both peptides are uncharged, it is conceivable that active transporters were involved in the relatively greater transport of Gly-Pro-Hyp. Overall, there is a paucity of research pertaining to BAP intestinal transporters, which requires more research using representative physiological models. Pro-Hyp has been shown to decrease the loss of chondrocytes, which synthesize articular cartilage [41]. In animal models designed to promote cartilage damage, Pro-Hyp inhibited cartilage thinning [41]. Accordingly, Pro-Hyp is considered to be one of the major bioactive components linked with the clinical efficacy of CHs towards treatment of osteoarthritis.

Our work assessing Hyp-Gly demonstrated transport (%) values of 62.41 ± 11.11 and 82.53 ± 36.53 for CH-GL and CH-OPT, respectively. Song et al. (2020) showed lower transport of Hyp-Gly (22.63 ± 5.19%) from silver carp skin hydrolysate after in vitro digestion and Caco-2 assessment using HPLC-ESI-MS analysis [7]. The greater degree of transport observed in our study may be attributed to the more physiologically relevant cell culture model used; the under expression of PepT1 in Caco-2 cells could significantly decrease the amount of peptide traveling across the intestinal layer. In contrast, the P_app_ values for Hyp-Gly (6.740 ± 1.200 × 10^−6^ after CH-GL and 5.593 ± 2.476 × 10^−6^ after CH-OPT) were lower compared to Song et al. (2020), which was 10.00 × 10^−6^ cm/s [7]. Apart from the different intestinal cell types used, variances in the quality of the established monolayer due to differences in passage number, cell conditions, and culture duration could impact the intestinal transport coefficients [42]. The high bioavailability of Hyp-Gly in the present work coincides with in vivo studies showing that this antiplatelet peptide is present in blood after CH ingestion and thereby could provide anti-thrombotic protection [7].

Although there were no differences in di-peptide bioavailability between the two tested CHs, CH-GL showed significant Gly-Pro-Hyp content after first pass liver metabolism, whereas none was observed after CH-OPT. This difference in bioavailability could be attributed to the presence of other peptides found within the CHs, as the digestion and bioavailability of BAPs can be affected by the presence of other peptides, proteins, or food components [2]. Increased peptide absorption could also occur due to synergisms with other peptides present in the digests as dietary AAs and protein hydrolysates can increase PepT1 expression [2]. Previous work by our group has established that CH-GL and CH-OPT have different peptide profiles, both pre- and post-digestion, with some peptide sequences being found in one CH and not the other [5]. The synergistic effects of BAPs are still under investigation; however, hormonal responses can be influenced by the presence of other proteins or peptides consumed. For example, the glucose-dependent insulinotropic polypeptide response and gastric emptying were greater when milk protein hydrolysates were ingested compared to whole milk protein sources [2]. Furthermore, colonic motility contractions were increased after whey hydrolysates compared to whey protein concentrates [2]. Further work on identifying and understanding synergistic effects affecting peptide transport, bioavailability and bioactivity, is required, particularly for CH-derived BAPs.

To our knowledge, the present study has been the first to determine the impact of hepatic first pass effects on BAPs after their intestinal transport. A direct and targeted method of BAPs quantification using CE allowed for an in-depth analysis of BAP content following their first pass effects. The presence of HepG2 cells in the basolateral compartment could potentially have affected permeability assessments, as previous work reporting P_app_ has used only intestinal cell monolayers. The effect of HepG2 cells in a co-culture on P_app_ has not been fully established. Some preliminary reports have demonstrated that the presence of Pro-Gly increases PepT1 expression in HepG2 cells [29], although further work is needed assessing peptide transport as affected by modulation of PepT1 expression by di-peptides. The use of a co-culture of intestinal and hepatic cell lines has been well established to understand bioavailability (%), although assessments of P_app_ were not reported [8,29,43]. Future work to incorporate hepatic effects on peptide transport should be investigated, especially considering that the expression of PepT1 may be regulated by the presence of BAPs [29].

The hepatic first pass effects on BAPs have not been well studied. Most published work discussed above investigating “bioavailability” only used Caco-2 cells thereby determining intestinal transport only, but this does not represent systemic availability. The degree that hepatic first pass effects affected peptide content in this study was unexpected; however, such studies investigating BAPs have not been previously performed. In that regard, it has been well established that there is high hepatic metabolism for small peptides [44], but hepatic upregulation of BAPs has not been studied previously. The importance of assessing the contribution of hepatic action is clearly demonstrated in our work. For example, Ala-Hyp was increased after incubating with HepG2 cells up to 304.9 ± 57.2% after treatment with CH-GL digests. Although both CHs were derived from bovine collagen, there was a significant difference in the hepatic first pass effects on Pro-Hyp. Hepatic action on Pro-Hyp was greater after CH-GL treatment (151.4 ± 24.3%) compared to CH-OPT (63.63 ± 8.63%); this was surprising as the content of Pro-Hyp that traversed across the intestinal layer was not significantly different between the treatments. The difference in hepatic first pass effects on Pro-Hyp might be due to the presence of Gly-Pro-Hyp that was solely noted to be intestinally transported after CH-GL treatment; this tri-peptide could conceivably be metabolized further by hepatic cells to contribute to the Pro-Hyp content. Such hepatic production of Pro-Hyp would not be expected with CH-OPT as Gly-Pro-Hyp was not appreciably transported across the intestinal layer with this treatment. The increase in BAP production for all the di-peptides during hepatic action could also have occurred due to the metabolism of unidentified longer chain peptides that travelled across the epithelium. In that respect, further work into identifying and assessing other collagen-derived BAPs is needed.

No previous studies have combined simulated digestion together with HIEC-6/HepG2-mediated transport and metabolism to investigate the bioavailability of CH-derived BAPs. A notable finding was that Gly-Pro-Hyp had a 12.24 ± 1.12% bioavailability with the CH-GL treatment after intestinal transport and hepatic first pass effects. A possible comparison might be made with the in vivo studies by Skov et al. (2019), which determined the postprandial plasma concentration of Gly-Pro-Hyp in a human clinical trial using ^1^H NMR analysis [4]. The initial Gly-Pro-Hyp content in the plasma was ~ 400 µM, and the Gly-Pro-Hyp content increased after 2 h to ~ 1050 µM, which would represent a 162.5% increase. It should be noted, however, that the method by which plasma Gly-Pro-Hyp was calculated by Skov et al. (2019), involved summing the individual AA measurements of Gly, Pro and Hyp, as no peptide sequencing or targeted quantification of Gly-Pro-Hyp was done. As digestion breaks down peptides into their AA components, it is possible that the summed plasma content of Gly, Pro, and Hyp indicated a greater apparent bioavailability of Gly-Pro-Hyp than provided via direct measurement of the tri-peptide.

To further understand the bioactivity of specific BAPs, rapid, accurate and efficient methods of identification and quantification are necessary. Previous work assessing CH-derived peptide bioavailability using Caco-2 cells have had significant limitations in terms of endpoint analysis. Feng et al. (2017) [19] assessed bovine CH bioavailability according to an indirect calculation of total AA transported. Furthermore, no peptide sequencing using proteomics methods or quantification was done. Three major AAs found in collagen are Gly, Pro and Hyp, but no Pro content was detected for all the hydrolysates assessed [19]; therefore, established BAPs sequences such as Pro-Hyp, Gly-Pro-Hyp, Gly-Pro, were likely not found. Future studies can utilize emerging technologies such as the CE methodology described herein towards the identification and quantitation of BAPs.

Despite their limitations, cell culture models continue to provide a platform to predict the bioavailability of BAPs, as animal studies often to do not correlate with human data, and human trials are long, associated with increased costs and have ethical restrictions [2]. Comparisons of cell culture models to human in vivo data generally support the use of the former to assess intestinal transport [22,23,24]. Discrepancies involving in vitro assessments of kinetics and peptide activity may occur, however, if the digestive and metabolic processes are not sufficiently considered [2]. Cell culture models must therefore accurately replicate the digestion, transport, and metabolism of the bioactive components of interest. For this reason, in this study, the bioavailability of CH-derived BAPs after in vitro digestion was determined using a novel co-culture of HIEC-6/HepG2 cells rather than a Caco-2 monolayer, as the expression of a key peptide transporter PepT1 is under-expressed in Caco-2 cells and predictions of peptide bioavailability could be misleading. Previous work has confirmed that HIEC cells more accurately represent the physiological in vivo conditions of the SI compared to Caco-2 cells [22,23,24]. Further studies can adopt and standardize this HIEC-6/HepG2 co-culture method, which could be adapted to investigate the first pass effects of bioactive food components, nutraceuticals and supplements.

As demonstrated in this study, similarly sourced and marketed CH products can contain different peptide profiles [5] and have varying degrees of peptide bioavailability. These findings are pertinent since BAPs must undergo first pass metabolism [9] for CHs and collagen-derived peptides to exert their bioactivity, such as on joint tissues including bone, cartilage and muscle. The bioavailability of collagen BAPs has been related to the clinically significant health benefits associated with CH intake, such as decreasing pain associated with OA, improving joint discomfort, and increasing bone mineral density [1,3,13,45]. Therefore, the different degree of BAP bioavailability seen after hepatic first pass effects between the CH products could modify their clinical efficacy. As consumers continue to use an increasing variety of over-the-counter CHs, assessing the bioavailability and bioactivity of BAPs from various CHs using higher throughput models is advantageous. This model provides a higher throughput method to assess peptide bioavailability before clinical studies are undertaken, which are often costly, long and have various ethical constraints.

## 5. Conclusions

The present study demonstrated the use of a more physiologically relevant model using a HIEC-6/HepG2 co-culture to assess the bioavailability of CH-derived BAPs after first pass metabolism. Furthermore, this study utilized an optimized CE method for the targeted assessment of BAPs from cell culture. Although both CHs were bovine sourced, differences in transport, hepatic effects and bioavailability were observed for different BAPs, which could potentially lead to different clinical results. Further clinical assessments of CHs are required to understand the impact of bioavailable BAPs. Overall, this study demonstrated a novel combination of techniques and cell lines that can be adapted to assess for the bioavailability of other drugs, nutraceuticals, and supplements, as well as their corresponding health promoting properties.

## Figures and Tables

**Table 1:** Peptide transport (%) from CH-GL and CH-OPT across intestinal epithelium.

	Peptide	Gly-Pro	Hyp-Gly	Ala-Hyp	Pro-Hyp	Gly-Pro-Hyp
Treatment						
CH-GL		33.11 ± 3.08	62.41 ± 11.11	9.27 ± 2.49	19.18 ± 4.81	59.44 ± 11.32
CH-OPT		40.35 ± 2.85	82.53 ± 36.53	26.4 ± 5.78	24.15 ± 1.42	nd

Values represent peptide concentration after transport (2 h timepoint) as a percentage of peptides of initial digesta values. For each peptide, a *t*-test was performed to determine differences in peptide transport between treatments, which were considered significant if *p* < 0.05. No significant differences in peptide transport were seen between treatments, however, no Gly-Pro-Hyp was detected in the basolateral compartment with CH-OPT (nd = not detectable).

**Table 2:** Hepatic effects on peptide content from CH-GL and CH-OPT following HepG2 incubation.

	Peptide	Gly-Pro	Hyp-Gly	Ala-Hyp	Pro-Hyp	Gly-Pro-Hyp
Treatment						
CH-GL		109.2 ± 9.600	55.16 ± 16.01	304.9 ± 57.2	151.4 ± 24.3 *	22.32 ± 5.09
CH-OPT		86.12 ± 14.09	28.23 ± 6.55	198.0 ± 107.6	63.63 ± 8.63	nd

Values represent peptide concentration after hepatic action (5 h timepoint) as a percentage of peptides available for HepG2 action (2 h timepoint). For each peptide, a *t*-test was completed to determine the effect of CH treatment, where differences were considered significant if *p* < 0.05. Asterisks represent significant differences between treatments (* *p* < 0.05), nd = not detectable.

## Supplementary Materials

The following are available online at https://www.mdpi.com/article/10.3390/cimb43030113/s1, Figure S1. Apparent permeability coefficient (P_app_) of CH-GL and CH-OPT peptides. Values are expressed as mean ± SEM in cm/s. For each peptide, a *t*-test was completed to determine the effect of CH treatment, where differences were considered significant if *p* < 0.05. Columns with asterisks are significantly different. Columns with ns are not significantly different.
