# Supplementary material for: Assessment of Bioavailability after In Vitro Digestion and First Pass Metabolism of Bioactive Peptides from Collagen Hydrolysates
Source: Curr Issues Mol Biol. 2021 Oct 13;43(3):1592–605. doi: 10.3390/cimb43030113 (PMC8928955; doi:10.3390/cimb43030113)
Supplement: Supplementary file 1 [file cimb-43-00113-s001.zip › Supplemental Figure S1.pdf]

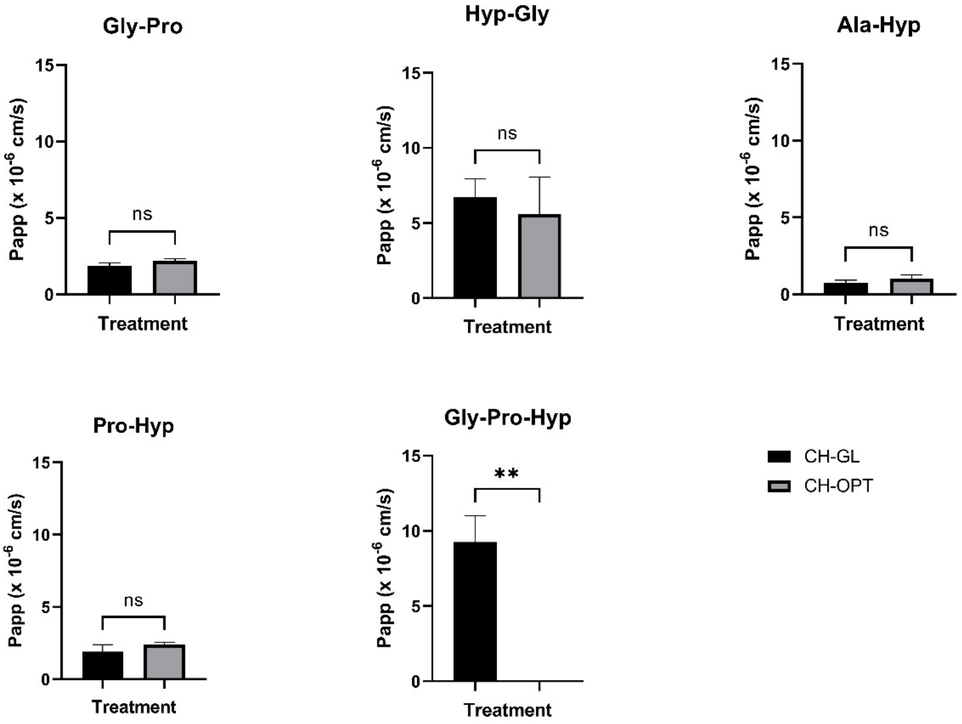

Figure S1. Apparent permeability coefficient (P_{app}) of CH-GL and CH-OPT peptides. Values are expressed as mean ± SEM in cm/s. For each peptide, a t-test was completed to determine the effect of CH treatment, where $p<0.05$ was considered significant. Columns with asterisks are significantly different (*** $p<0.001$). Columns with ns are not significant.
